# Maculopapular Cutaneous Mastocytosis in a Six-month-old Boy Who Presented with Respiratory Distress

**DOI:** 10.7759/cureus.4117

**Published:** 2019-02-22

**Authors:** Maira Abdul Razzak, Laila Tul Qadar, Maria Khan, Mohammad Hasan, Ammarah Jamal

**Affiliations:** 1 Pediatrics, Civil Hospital Karachi, Karachi, PAK; 2 Internal Medicine, Dow University of Health Sciences, Karachi, PAK; 3 Internal Medicine, Jinnah Sindh Medical University, Karachi, PAK

**Keywords:** maculopapular cutaneous mastocytosis, respiratory distress, skin, pakistan

## Abstract

Mastocytosis is a diverse group of rare disorders characterized by mast cell proliferation and its aberrant accumulation within various organs including respiratory, gastrointestinal, genitourinary mucosa and most commonly the skin. The spectrum of presentations ranges from torpid disease (cutaneous mastocytosis) having normal life span with transient sign and symptoms to highly vigorous disease (systemic mastocytosis) and life-threatening malignant conditions. Prevalence of the disease in general population is unknown. It occurs in all races and there is no sex predilection. Peak incidence is during infancy and early childhood with second peak occurring in middle age. We report a case of maculopapular cutaneous mastocytosis (MCM) in a six-month-old male child who presented with respiratory distress. According to our literature search it is one of the least frequent cases reported in our country, Pakistan.

## Introduction

Mast cells are biologically active cells containing histamine and heparin, which are liberated in response to emotional stress, physical stimuli (e.g., heat, cold, friction, exercise), bacterial toxins, venom, immunologic stimuli (e.g., IgE), polymeric eye drops (containing dextran), complement-derived anaphylotoxins, drugs, etc. Mastocytosis results when mast cells proliferate with consequent aberrant collection within dermis predominantly within respiratory, gastrointestinal, and genitourinary mucosa [1]. Mastocytosis refers to a diverse group of disorders with spectrum of presentations ranging from torpid disease [cutaneous mastocytosis (CM)] with normal life span to highly vigorous disease (systemic mastocytosis) and malignant conditions. Clinical categories of CM include maculopapular cutaneous mastocytosis (MCM) which is also known as utricaria pigmentosa, diffuse cutaneous mastocytosis, and solitary cutaneous mastocytoma in order of decreasing frequency [2]. Firm rubbing of the skin lesions affected by mastocytosis forms a diagnostic wheal, Darier’s sign [3] which is pathognomic of this disorder. Studies suggest that in about 75% of cases, peak incidence of mastocytosis occurs in first two years of life [4]. We report a rare case of MCM in a six-month-old boy who presented with respiratory distress.

## Case presentation

**Figure 1:**
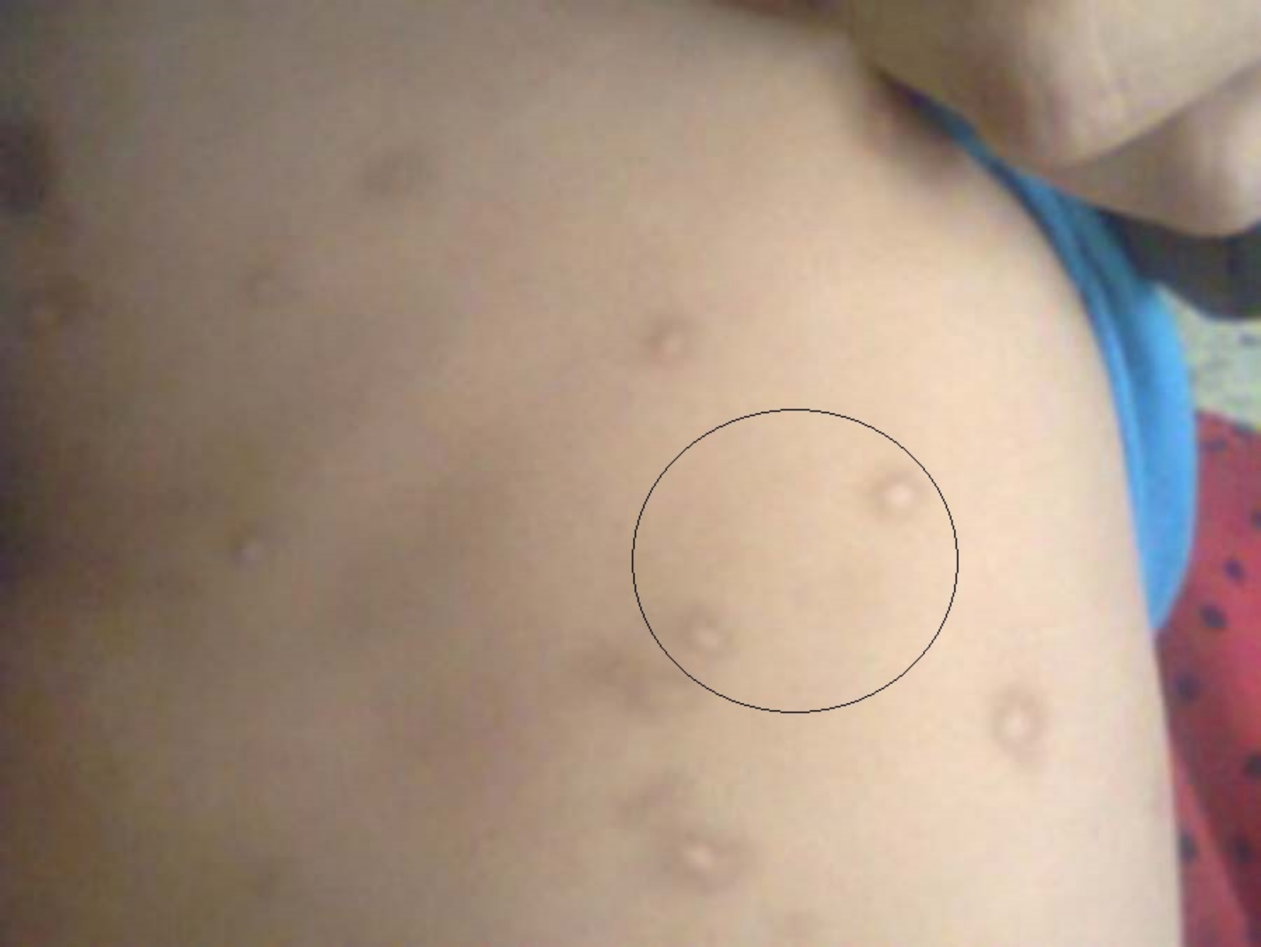
Maculopapular cutaneous mastocytosis nodules on trunk region.

A six-month-old, exclusively breastfed male, weighing 7.1 kg was admitted to the pediatric ward of Dr. Ruth KM Pfau, Civil Hospital Karachi (CHK) with a three-month history of nodules and a two-day history of respiratory distress. The patient was asymptomatic three months back, when his mother noted formation of multiple nodules on his trunk (Figure 1) and face. It was initially diagnosed as nodular scabies by a dermatologist because of its high frequency in our community and similar rash. Anti-scabies treatment was prescribed but as the number of nodules increased to involve the entire body including both extensor and flexor surfaces, he was hospitalized for evaluation. Presence of nodules was not associated with joint pains, vomiting, or diarrhea. There was no family history of similar disease. Other differential considered now for the skin lesions was one of the varieties of histocytosis type IIa including juvenile xanthogranuloma, xanthoma disseminatum, and progressive nodular histocytosis.

On examination, at the time of admission, patient was irritable showing signs of respiratory distress. He had a temperature of 101°F, respiratory rate of 60 beats/min, heart rate of 110 beats/min, and oxygen saturation of 96% with normal anthropometry. Auscultation of the chest revealed bilateral expiratory wheezes all over the chest. He also had multiple oval nodules all over his body, varying in size from 1 to 1.5 cm, discrete, firm, erythematous, and nontender with hyperpigmented margins and smooth surface. They were mostly distributed on trunk and face. On stroking the individual lesion, there was formation of wheal and erythema (Positive Darier’s Sign) thus suggesting the diagnosis of mastocytosis.

 Laboratory investigations showed an almost normal complete blood count (CBC) with hemoglobin of 10 g/dL, mean corpuscular volume (MCV) of 71.2 fl, mean corpuscular hemoglobin (MCH) of 25.3 pg, and mean corpuscular hemoglobin concentration (MCHC) of 35.5 g/dl. Hematocrit (Hct) was 26.2%, total leucocyte count (TLC) was 10,000cells/cumm, and platelet count was 210,000/µmL. He had a normal blood urea nitrogen (BUN) of 5 dl/L, a normal serum creatinine of 0.2 mg/dL, and normal serum electrolytes with sodium of 139 mEq/L, potassium of 4.7 mEq/L, chlorine of 104 mEq/L. Serum tryptase and serum and urine histamine levels were also performed and found to be within normal range thus ruling out the possibility of systemic mastocytosis. To confirm the diagnosis of mastocytosis it was decided in consultation with the dermatologist to go for the histopathological examination of the nodule and thus biopsy of the nodule was performed. Histopathological examination showed diffuse infiltration of mast cells in the form of a broad band under the epidermis, depicting consistency with mastocytosis. Patient was put on oral prednisolone in a dose of 2 mg/kg once daily for four weeks, which improved the child’s condition with regression of the nodules. After steroids prescription multiple follow-up has been done and the child is still in remission. Parents were counseled to avoid scrubbing and massaging of the skin which may lead to worsening of the lesions.

**Figure 2:**
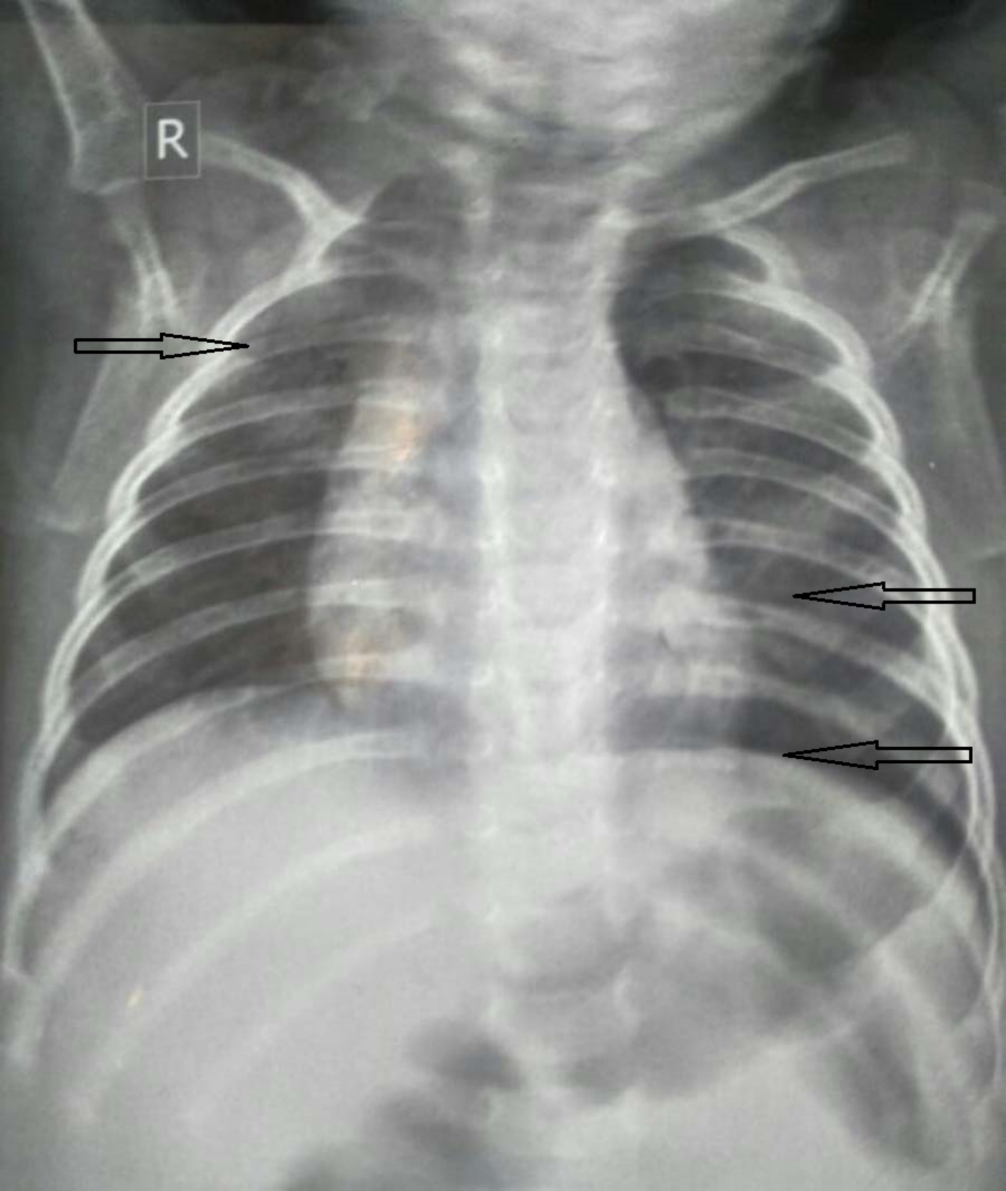
Chest X-ray representing hyperinflation of lungs with flattening of the diaphragm and increased hilar bronchial markings, suggestive of bronchiolitis.

Chest X-ray, which was done for his respiratory symptoms revealed hyperinflation of lungs with flattening of the diaphragm, horizontal ribs, and increased hilar bronchial markings (Figure 2). On the basis of his respiratory findings and chest X-ray the patient was diagnosed as having bronchiolitis and was treated with antipyretic and bronchodilator therapy using ipratropium bromide ( 0.25 mg NEB q20min x three doses) nebulization initially, followed by oral terbutaline which resulted in subsequent improvement in his respiratory symptoms within four days. Patient is doing well two weeks post discharge.

## Discussion

Mastocytosis is mainly a sporadic disease; however, familial cases are also reported in literature [5]. Mast cells express a cell surface receptor, proto-oncogene c-kit (CD117), which is the receptor for stem cell factor (SCF) and appears to be important for the proliferation of mast cells. Mutations of the c-kit receptor, lead to uncontrolled stimulation of the receptor and play an important role in disease pathogenesis. Bodemer et al.’s research depicted series of childhood cases of mastocytosis in which there was an elevating rate of somatic mutation leading to c-KIT activation in their pediatric population [6]. Mastocytosis disease in childhood is linked to Glu-839-Lyc c-kit mutation [5]. Genetic workup was not performed due to its unavailability in our healthcare institute leading to one of the limitations of our report.

Cutaneous mastocytosis can easily be mistaken for a variety of similar rashes that plague the pediatric population such as scabies. In our case we suspected mastocytosis based on the specific nodular lesions and hyperpigmented margins with positive Darier’s sign. According to a study conducted by Kiszewski et al., a total of 71 children with CM were evaluated in a year in which 92% showed disease onset within first year of life and displayed a male predominance. Moreover, 94% cases of the same study exhibited Darier’s sign [7]. The age and gender of our case subject along with the positive Darier’s sign also conforms to the above-mentioned study [5].

Another series which reported six cases of mastocytosis by Inamadar and Palit showed substantial response to systemic steroids [8]. In this regard, we had a similar experience. Systemic steroids brought rapid and long-lasting remission. Correia et al.’s study of a 14- and 26-month-old male children established that usage of pimecrolimus cream twice a day with oral antihistamine use for three months showed effective results in the patients without any clinical evidence of recurrence [9]. Similarly according to a recent study, application of topical pimecrolimus 1% in pediatric population of CM efficiently wiped out 3/4th of the lesions without resulting in any systemic or topical complications [10]. Hence, in treatment of CM, topical therapy with 1% pimecrolimus should be taken into consideration.

After a thorough literature review we found that CM is one of the least reported cases in infants, so it might be the first case report highlighting the MCM incidence in infant population of Pakistan.

## Conclusions

The purpose of reporting this case is to highlight the case of MCM which is commonly misdiagnosed as scabies; hence pediatricians and dermatologists should remain aware of varied forms of CM because of its rarity and the distinctive management of each individual case which needs confirmation by histopathological examination. Our patient presented with respiratory distress which was diagnosed and treated as bronchiolitis and considered as a coincidental illness. Whether the respiratory distress can be a part of mastocytosis because of respiratory involvement remains to be seen.
